# Literature optimized integration of gene expression for organ-specific evaluation of toxicogenomics datasets

**DOI:** 10.1371/journal.pone.0210467

**Published:** 2019-01-14

**Authors:** Katerina Taškova, Jean-Fred Fontaine, Ralf Mrowka, Miguel A. Andrade-Navarro

**Affiliations:** 1 Faculty of Biology, Biozentrum I, Mainz, Germany; 2 Experimentelle Nephrologie, Universitätsklinikum Jena, KIM III, Jena, Germany; Institut de Pharmacologie Moleculaire et Cellulaire, FRANCE

## Abstract

The study of drug toxicity in human organs is complicated by their complex inter-relations and by the obvious difficulty to testing drug effects on biologically relevant material. Animal models and human cell cultures offer alternatives for systematic and large-scale profiling of drug effects on gene expression level, as typically found in the so-called toxicogenomics datasets. However, the complexity of these data, which includes variable drug doses, time points, and experimental setups, makes it difficult to choose and integrate the data, and to evaluate the appropriateness of one or another model system to study drug toxicity (of particular drugs) of particular human organs. Here, we define a protocol to integrate drug-wise rankings of gene expression changes in toxicogenomics data, which we apply to the TG-GATEs dataset, to prioritize genes for association to drug toxicity in liver or kidney. Contrast of the results with sets of known human genes associated to drug toxicity in the literature allows to compare different rank aggregation approaches for the task at hand. Collectively, ranks from multiple models point to genes not previously associated to toxicity, notably, the PCNA clamp associated factor (PCLAF), and genes regulated by the master regulator of the antioxidant response NFE2L2, such as NQO1 and SRXN1. In addition, comparing gene ranks from different models allowed us to evaluate striking differences in terms of toxicity-associated genes between human and rat hepatocytes or between rat liver and rat hepatocytes. We interpret these results to point to the different molecular functions associated to organ toxicity that are best described by each model. We conclude that the expected production of toxicogenomics panels with larger numbers of drugs and models, in combination with the ongoing increase of the experimental literature in organ toxicity, will lead to increasingly better associations of genes for organism toxicity.

## Introduction

Therapeutic drugs, industrial and environmental chemicals, and other compounds can have undesired toxic effects in diverse parts of the human body. This might be due to their direct interaction with cell components, but also, if they are metabolized, due to effects of their downstream catabolism [1]. These direct and indirect effects depend on the tissues considered (e.g., hepatotoxicity in liver [2] or nephrotoxicity in kidney [3]), on the human individual genetic variation (e.g., if a catabolizing enzyme has variable specificity associated to a single nucleotide polymorphism [4]) and on environmental factors (e.g., interaction with nutrients [5]). Thus, the toxic effects a given compound exerts in a given organ are a result of very complex interactions at cellular, organismic and environmental level, involving often multiple toxicity mechanisms [6].

The organ-specific study of drug effects on human physiology, while necessary, is however difficult given the impossibility of systematically testing the human system. Therefore, the commonly established approaches in the field of drug development and toxicology rely on studies of drug effects in animal organisms (*in vivo*) or in cultivated animal and human cell lines (*in vitro*). Profiling of gene expression can inform at a very detailed level about the effects of drugs. With the advent of high throughput technologies (in particular, the cost-effective microarrays), genome-wide transcription profiles of specific drug effects have been captured across multiple organs or cell types, drug dose levels and drug treatment time intervals. This enabled toxicologists to study the biochemical and biological mechanisms affected by a specific drug as well as the mechanisms behind organ-specific and general drug-induced toxicity. Most importantly, large-scale initiatives (e.g., DrugMatrix by Iconix Biosciences [7], the Japanese toxicogenomics project [8] and the EU funded InnoMed PredTox project [9]) have produced highly controlled and standardized toxicological experiments with rodent organisms (rat and mouse) and rodent and human cell lines, for hundreds of chemicals, resulting in comprehensive toxicogenomics resources for systematic analysis of drug induced toxicity effects, such as DrugMatrix [7], CMap [10] and Open TG-GATEs [11].

In this respect, the aim of the recently established field of toxicogenomics is to integrate these rapidly expanding collections of data (including gene expression profiles, clinical chemistry measurements, histopathology assessment data and curated literature annotations) in order to identify more specific and sensitive human toxicity biomarkers that should lead to more accurate toxicity detection at early stage of drug development and ultimately to safe marketed drugs [12].

While their contribution to our understanding of drug-induced toxicity is undeniable, one fundamental question that is difficult to address comprehensibly regarding the different systems used for experimentation in toxicogenomics studies is how reliably the specific animal organism, model system (*in vivo* or *in vitro*), reflects the toxicity effects of drugs for a given human organ in terms of genes (or molecular functions) whose expression is affected. In particular, it would be desirable to have a protocol to evaluate the appropriateness of multiple model systems so that the optimal one can be chosen for each organ, depending on the ability of the given model to affect the expression of genes and functions of interest.

Here, we describe a method to obtain lists of ranked genes according to gene expression changes in a toxicogenomics dataset profiling gene expression changes upon multiple drugs, conditions, and time points. If the dataset uses a particular tissue or cellular line as model to test drug toxicity, the expectation is that the ranked list reflects genes generally associated to toxicity in this model. Because our goal is to model toxicity in human organs, if the model is non-human, we take the corresponding human orthologous genes. Then, we optimize the methodology and parameters to obtain this ranking by comparison to the set of genes already known to be associated to drug toxicity in the corresponding organ in the literature.

Due to the multi-factorial experimental designs of the existing toxicological databases, we need to integrate data across multiple model systems, drugs, dose levels and time points. A relatively simple way is to summarize the genome-wide drug-induced transcriptional changes in prioritized (ranked) gene lists, followed by a rank-aggregation approach [13] to combine them into a single organ-specific gene list. While this approach is commonly used for prioritization of disease-related genes [14], here we use it as baseline method for prioritization of toxicity-related genes. In particular, we exploit the robustness (with respect to noise, outliers and errors in the data to be aggregated) and computational efficiency of recently proposed probabilistic methods for rank-aggregation, i.e., RRA [15] and BIRRA [16].

We applied this approach to a toxicogenomics dataset corresponding to 33 compounds (mainly toxic drugs) profiled in primary human hepatocytes, primary rat hepatocytes, rat liver (as models for hepatotoxicity) and rat kidney (as model for nephrotoxicity), obtained from the Open TG-GATEs database. The latter was especially suitable for our purposes due to the consistent experimental design applied across the four model systems. We evaluated multiple ways to aggregate these data for the description of drug-induced toxicity in human kidney and liver.

In doing this analysis, we discovered some genes that while ranking very high and very often in response to liver and kidney drug toxicity, have not been yet mentioned in the literature in relation to toxicity. These could be potentially working in novel toxicity-related pathways. We discuss the striking differences observed between human and rat hepatocytes, and the insight that can be gained into which processes can be modelled when using cell lines (rat hepatocytes) versus entire organs (rat liver). We use these results to describe the suitability of each of these models to study different molecular functions associated to organ toxicity according to the sets of genes whose expression is affected.

## Methods

Unless otherwise stated, the complete protocol for data analysis was performed using the R version 3.2 computing environment [17] and packages from the open-source software development project Bioconductor version 3.1 [18].

### Toxicogenomics data collection

The data used in this study originate from the Open Toxicogenomics Project-Genomics Assisted Toxicity Evaluation System (TG-GATEs) database established during the Japanese Toxicogenomics Project [8]. This large-scale public toxicogenomic resource stores microarray gene expression and toxicological profiles of *in vivo* (rat liver and kidney) and *in vitro* (primary cultured rat and human hepatocytes) biological samples exposed to 170 different chemical compounds [11]. The experimental design involved at least two biological replicates per experiment (three in the *in vivo* experiments), compound administration at different dose levels (low, middle and high, as well as a vehicle control corresponding to no compound administration) and time measurements taken in two different scenarios (single-dose and repeated-dose). The single-dose scenario included up to four measurements taken after single compound dose administration (at 2, 8 and 24 hours in the *in vitro* and at 3, 9, 12 and 24 hours in the *in vivo* experiments, respectively); the repeated-dose scenario included single dose administration per day per rat, and measurements were taken at 4, 8, 15 and 29 days. The gene expression of the obtained samples was profiled with two Affymetrix GeneChip arrays: Rat Genome 230 2.0 (rat samples) and Human Genome U133 Plus 2.0 (human samples).

In order to have fair comparison across the model systems, we used the gene expression profiles corresponding to a subset of 33 compounds, with a compound being selected if it was used in the single-dose experiments across all model systems, i.e., human hepatocytes (HH), rat hepatocytes (RH), rat liver (RL) and rat kidney (RK). Among the selected compounds (listed in S1 File), we found 26 drugs with diverse therapeutic use (e.g., the widely used analgesic acetaminophen, the antifungal ketaconazole and the antibacterial rifampicin) and 7 environmental toxicants (allyl alcohol, bromobenzene, bromoethylamine, ethionine, hexachlorobenzene, monocrotaline, and thioacetamide). Except for caffeine, all compounds are classified in the literature as hepatotoxic or nephrotoxic (including the ones being carcinogenic for the liver or the kidney), with 20 compounds harmful for both kidney and liver (e.g., the gout-related agent allopurinol, the anti-inflammatory indomethacin, the antibacterial nitrofurantoin and the carcinogenic thioacetamide). While the majority of drugs belong to the enzyme inhibitor class in terms of mode of action (that defines how the compound affects the molecular target), these compounds are more diverse in terms of physiochemical properties. More precisely, only acetaminophen, bucetin and phenacetin cluster together after binning clustering with 0.4 cut-off for compound similarity in the web tool ChemMine [19]. The compound similarity was defined by the Tanimoto similarity coefficient, also known as Jaccard index [20], and calculated using the Open Babel molecular descriptors for the compounds. The latter were calculated also in ChemMine based on the compound structure representations in simplified molecular input line entry system (SMILES) as provided in S1 File.

While the full experimental design is available for the rat samples, gene expression profiles for 15 compounds were not conducted for the low dose level or for the earliest time point in HH. The raw gene expression data in .CEL Affymetrix file format and the corresponding metadata file (with the GeneChip code, experiment-description features and toxicological data) were downloaded from the archived site [21].

### Microarray data preprocessing

The .CEL files corresponding to the selected compound treated samples were processed in four batches with 623 HH, 792 RH, 1584 RL and 1584 RK samples, respectively. The probe intensity data were mapped to probesets expression data with the Robust Multiarray Average (RMA) algorithm [22], which combines convolution background correction, quantile normalization and median-polish-based multiarray summarization, as implemented in the package affy version 1.46.1. This step delivered estimate expression values for 54,675 and 31,099 probesets per profiled sample with the Human Genome U133 Plus 2.0 and the Rat Genome 230 2.0 arrays, respectively. These were furthermore filtered to remove non-specific probesets mapping to multiple gene NCBI Entrez IDs, and to select a single probeset per gene. The latter was done by choosing the most variant probeset across all model system-specific conditions whenever multiple probesets mapped to one gene. The most variant probeset was defined to be the one with the largest interquartile range of expression values across all samples for a given model system, possibly the most informative. While this approach can be problematic when probesets represent multiple gene transcripts that are expressed at very different levels, it is commonly used as it provides a single result per gene. This step resulted in 20,390 human probesets, and 14,517 rat probesets annotated with a unique NCBI Entrez ID. Probesets’ annotations with gene symbols and IDs were obtained with the corresponding metadata packages: hgu133plus2.db version 3.1.3 (Human Genome U133 Plus 2.0 arrays) and rat2302.db version 3.1.3 (Rat Genome 230 2.0 arrays).

### Differential expression analysis

For each model system, we performed compound-wise analysis of differential expression (DE) with the Microarray Significant Profiles (maSigPro) method, implemented in the package maSigPro version 1.40.0 [23]. maSigPro is a two-step regression method that models the dynamic nature of gene expression by fitting a global polynomial model of time with dummy variables used to encode different experimental groups. The first regression step essentially finds statistically significant gene models, i.e., genes that show change over time in at least one experimental group (including the control group) or treatment-dependent expression. The second step uses stepwise regression to adjust the model coefficients of the significant gene models in order to find significant differences among the experimental groups, in particular among the treatment and the control groups. We selected differentially expressed genes with the following parameter setup: (i) global polynomial model with up to cubic time effect (due to the four time measurements) and three dummy variables (one for each dose level), (ii) P-value ≤ 0.01, 0.01 cut-off for the P-value associated to the F-test statistics in the global model, after adjustment with the false discovery rate (FDR) method by Benjamini and Hochberg [24], (iii) two-way backward stepwise regression with P-value ≤ 0.05/n (coefficient-related P-value in the adjusted models, with n being number of model coefficients), and (iv) R2 ≥ 0.6, at least 60% of gene expression variance explained by the adjusted gene model.

Genes selected by this method are the ones that show variations in differential expression (versus control cases) over time. We applied ARSyN noise removal on the compound-specific expression matrix before applying maSigPro following the strategy suggested by Nueda et al. [25].

### Gene ranking

To enable cross-organism comparison we analyzed the differential gene expression of the 12,037 protein-coding genes that have a one-to-one mapping between human and rat as defined in the NCBI HomoloGene version 68 database [26].

The genes profiled on the human or rat arrays were ranked independently for each compound and model system according to the results from maSigPro: (i) all genes that did not pass the F-test were given the same lowest rank, (ii) the rest were ranked by R2 of the adjusted model with ties resolved by the P-value of the adjusted model, and (iii) the genes that after model adjustment had no significant model coefficient were also given the same lowest rank. The resulting ranking is technically an extended partial ranking.

We applied several rank aggregation methods to obtain a single rank for each of the 12,037 genes across all 33 compounds in each given model system.

#### Probabilistic methods used for rank aggregation

Robust Rank Aggregation (RRA) is a parameter-free method that provides a significance score (P-value) for the calculated rank [15]. Assuming the genes of interest will be highly ranked across many toxic compounds (informative ranks) and ignoring the noise from the small portion of non-toxic compounds (non-informative ranks, including possible outliers), RRA models the informative normalized ranks as sampled from a zero-skewed distribution. It calculates a score for a single gene at a time, by comparing the sorted compound-specific normalized ranks for the given gene to a fixed null model of uniformly-random distributed normalized ranks. The latter results in the assignment of a P-value for each sorted compound-specific rank, and the minimal P-value across all compounds (after Bonferroni adjustment for multiple comparisons across compounds) is used as the gene-associated score. This score is actually used to re-rank all the input genes and at the same time it represents a significance score (P-value) showing how much better a gene was ranked than expected by chance. STUART, the predecessor of RRA, is less computationally efficient and does not provide rank significance [14, 27]. Bayesian Inference Robust Rank Aggregation (BIRRA) implements rank aggregation via Bayesian reasoning [16]. The method assumes that there is a latent signal to be discovered and this is represented with varying reliability (weight) in the datasets to be integrated. In our case, for example, gene lists from toxic compounds should contribute more (relative to the non-toxic compounds) to the aggregated list, assuming there exist (hidden) toxicity-related traits that are contributed by a common set of genes across organ-specific toxic compounds. The weights are directly estimated from the data in the form of Bayes factors: starting with an equally weighted consensus generated by rank averaging, BIRRA iteratively fits Bayes factors to each dataset and recomputes the rank aggregation until either there are no changes in the rankings or the maximum number of iterations is exceeded.

#### Ad-hoc rank methods used for comparison

We further used aggregation based on simple average of ranks (MEAN), rank by the number of compounds that modulated the gene (NC), and finally, the median of the aggregated ranks RRA, MEAN, STUART, BIRRA, and NC (AR).

We used RRA and STUART as implemented in the package RobustRankAgregg version 1.1. The same also implements the significance score for the MEAN rank. The MEAN/RRA P-values were additionally corrected for multiple testing with the FDR method by Benjamini and Hochberg [24] before using them for gene ranking. The R source for BIRRA was downloaded from the author’s site [28] and was applied with default parameter values (number of bins set to 50, maximum number of iterations set to 10 and prior probability of the positive class set to 0.05). Finally, all tie cases (same value of the ranking criteria) were resolved by assigning equal rank calculated as average of the default-assigned ranks by the ordering procedure.

### Toxicity-related genes from the literature

To evaluate and optimize the ranks for the prioritization of toxicity relevant genes from the toxicogenomics dataset we collected genes discussed in the literature in relation to organ-specific (liver or kidney) toxicity.

The publicly available PubMed annotations [26] were used for prioritizing gene candidates related to the topics of liver or kidney-related human toxicity. This was performed as a gene-topic co-occurrence analysis in PubMed abstracts (see below for details). The statistical significance of each gene-topic association was measured by the one-tailed Fisher’s exact test [29]. Only human protein-coding genes were tested. The background set was the same in all tests, and was defined by the complete set of PubMed abstracts written in English. The subset of abstracts corresponding to each human gene (manually annotated by NCBI curators) was extracted from gene2pubmed and GeneRIF files [30]. Furthermore, the toxicity-related abstracts were filtered by organ and defined as the set of PubMed abstracts obtained by the following PubMed search queries: (i) “nephrotoxicity OR [toxicity AND kidney]”, and (ii) “hepatotoxicity OR [toxicity AND liver]”. Note that the queries aim to capture all abstracts related to organ-specific toxicity and not only drug-induced organ-specific toxicity.

Gene-topic association-wise Fisher’s exact test statistics were adjusted with the FDR method by Benjamini and Hochberg [24]. Genes with topic-association both under the 0.05 cut-off and supported with at least three PubMed abstracts were selected as literature candidates. After subsequent filtering for genes that have a one-to-one rat ortholog in the NCBI HomoloGene v.68 database [26], we finally obtained 48 and 21 literature gene candidates for hepatotoxicity and nephrotoxicity, respectively. Both lists were subjected to disease enrichment analysis to verify the biological relevance for human toxicity. This was performed with the Gene Set to Diseases (GS2D) web tool [31]. The results were filtered at 0.05 FDR.

### ROC analysis

To evaluate the rankings obtained by the different methods for toxicity gene prioritization we calculated the receiver-operating characteristic (ROC) curve and the Area Under the ROC Curve (AUC), using the literature-derived toxicity genes as positive examples and all the remaining genes from the full list of ranked genes as negative examples. Calculations were performed with the package pROC version 1.8 [32].

### Rank correlation analysis

We used Spearman’s rho, a nonparametric correlation coefficient, to assess statistical associations between two lists with gene-wise ranks. Spearman’s rho has values between -1 and 1, with positive correlation values indicating similar (or identical for correlation of 1) gene ranks in both lists, while negative values indicate dissimilar (or fully opposed for a correlation of -1) ranks in both lists. The correlation values, including the test for no association, were calculated with the package stats version 3.2.3 [17].

### Visualization

Most of the figures were generated with the packages ggplot2 version 2.1.0 [33] and graphics version 3.2.3 [17]. The plots visualizing the modules and their overlaps are generated with the customized heatmap function [34], while module regulatory networks were visualized with GePS.

### Functional enrichment analyses

To assess the significance of functional enrichment in ranked lists of genes, we used the g:Profiler web tool (here, with the top 100 genes; [35]). This tool computes the over-representation of functional terms in the ranked list of genes. The terms assessed were from the Gene Ontology (GO) hierarchy: biological process (BP), molecular function (MF) and cellular components (CC), and we also considered terms from the KEGG (keg) and REACTOME (rea) pathway databases, CORUM protein complexes (cor), TRANSFAC binding sites (tf), and Human Protein Atlas (hpa). GO terms were hierarchically filtered to best per parent.

## Results

To develop a protocol for gene prioritization according to organ-specific toxicity from large-scale panels of toxicogenomics profiles, we integrated microarray gene expression time series from the Open TG-GATEs database [11] corresponding to 33 compounds (the majority of which are nephrotoxic or hepatotoxic drugs, S1 File) for four model systems: human hepatocytes (HH), rat hepatocytes (RH), rat liver (RL) and rat kidney (RK). We chose to analyze HH, RH, and RL to be able to compare multiple models for hepatotoxicity (*in vivo* versus *in vitro*, and in human versus rat), and then added RK as model for nephrotoxicity, to be able to compare results for two different *in vivo* models (liver versus kidney).

After microarray preprocessing and gene expression modeling, we performed a differential analysis to identify genes with significant differential expression profiles over time for each compound and model system. This step was crucial for the protocol as it provides the compound-specific gene ranks that serve as input for the rank-aggregation step.

Using different rank-aggregation methods, compound-wise gene rankings were summarized into one ranked gene list, which was limited to one-to-one orthologs between human and rat (see Methods, subsection **Gene ranking** for details). We evaluated the performance of the rank-aggregation methods against human liver and kidney toxicity-related genes derived from co-occurrence analysis of PubMed abstract annotations (see Methods, subsection **Toxicity-related genes from the literature** for details). We further compared the rank-aggregation methods based on the receiver-operating characteristic (ROC) curve analysis, in order to select the rank aggregation that shows better concordance with literature-derived toxicity genes (see Methods, subsection **ROC analysis** for details). The optimal rank aggregation was used to generate four gene candidate lists, one for each model system, which were finally subjected to functional enrichment analysis to evaluate the ability of the four models (HH, RH, RL, RK) to recapitulate human organ-specific drug toxicity.

### Toxicity-related genes from literature

Based on 70,190 and 39,365 PubMed abstracts for liver and kidney toxicity, respectively, we found 65 and 28 human protein-coding genes significantly associated with at least three toxicity-related abstracts at 0.05 FDR, with ten genes common for both organs. Selection of genes with orthologs in rat resulted in 48 and 21 genes for liver and kidney toxicity, respectively (see Methods for details). Associations of these genes to disease related terms in the literature were found to be significantly enriched in terms describing organ-specific injuries, such as acute kidney injury, glomerulonephritis, renal insufficiency as well as drug-induced liver injury and hepatocellular carcinoma. The hepatic toxicity list includes hepatotoxicity biomarkers such as heme oxygenase (decycling) 1 (HMOX1), ATP binding cassette subfamily C member 2 (ABBC2), glutathione S-transferase alpha 1 and pi 1 (GSTA1 and GSTP1), keratin 18 (KRT18) [36, 37]. The kidney toxicity list includes nephrotoxicity biomarkers such as kidney injury molecule-1 (KIM1 or HAVCR1), lipocalin 2 (LCN2), and solute carrier family 22 member 6 (SLC22A6) [38]. The lists of genes (names and Entrez IDs) including PubMed IDs (PMIDs) of the contributing abstracts are provided in S2 File.

### Differential gene expression analysis

**Fig 1:**
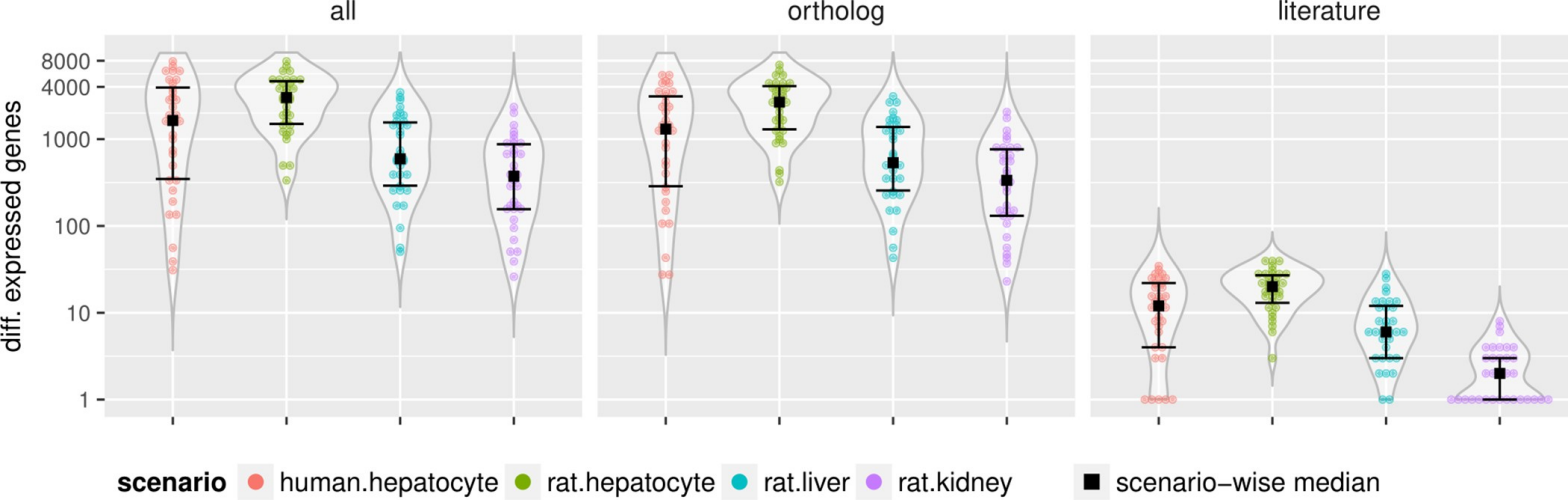
Summary of differential expression analysis. Distribution of the number of differentially expressed genes is depicted with violin-plots by model systems, with each dot representing the number of genes modulated by the high dose treatment with a single compound. The median and interquartile range of each distribution is depicted by black squares and black bars, respectively. On the same plot, we illustrate the distribution of the full set (all), the filtered set with unique human-rat orthologs (ortholog) and the subset found in the corresponding organ-specific literature-derived genes (literature).

The overall analysis across the four model systems shows that the literature-derived toxicity-related human genes are more often dys-regulated in the *in vitro* scenarios as compared to the *in vivo* ones (Fig 1). On average, we have far more genes dys-regulated in both *in vitro* systems (HH and RH) as compared to both *in vivo* systems (RL and RK), which might be a consequence of isolation stress or because of the cell culture conditions as noted before by Godoy et al. [39].

Furthermore, we observe a large discrepancy in the number of differentially expressed genes across compounds, which shows good correlation with the increase of dose level (S1 Fig), and in many cases, also with the known toxicity label of the drug. For example, indomethacin is a known toxicant with high liver injury risk and shows high number of differentially expressed genes in the hepatocyte and liver systems (S2 Fig). The same holds for the hepatotoxic analgesic acetaminophen (commonly known as paracetamol), although we observe an inconsistent pattern as well, e.g., known hepatotoxic drugs such as enalapril and valproic acid dys-regulated ten (or more) times less genes in the RL than in the RH system.

### Rank aggregation across compounds and scenarios

**Fig 2:**
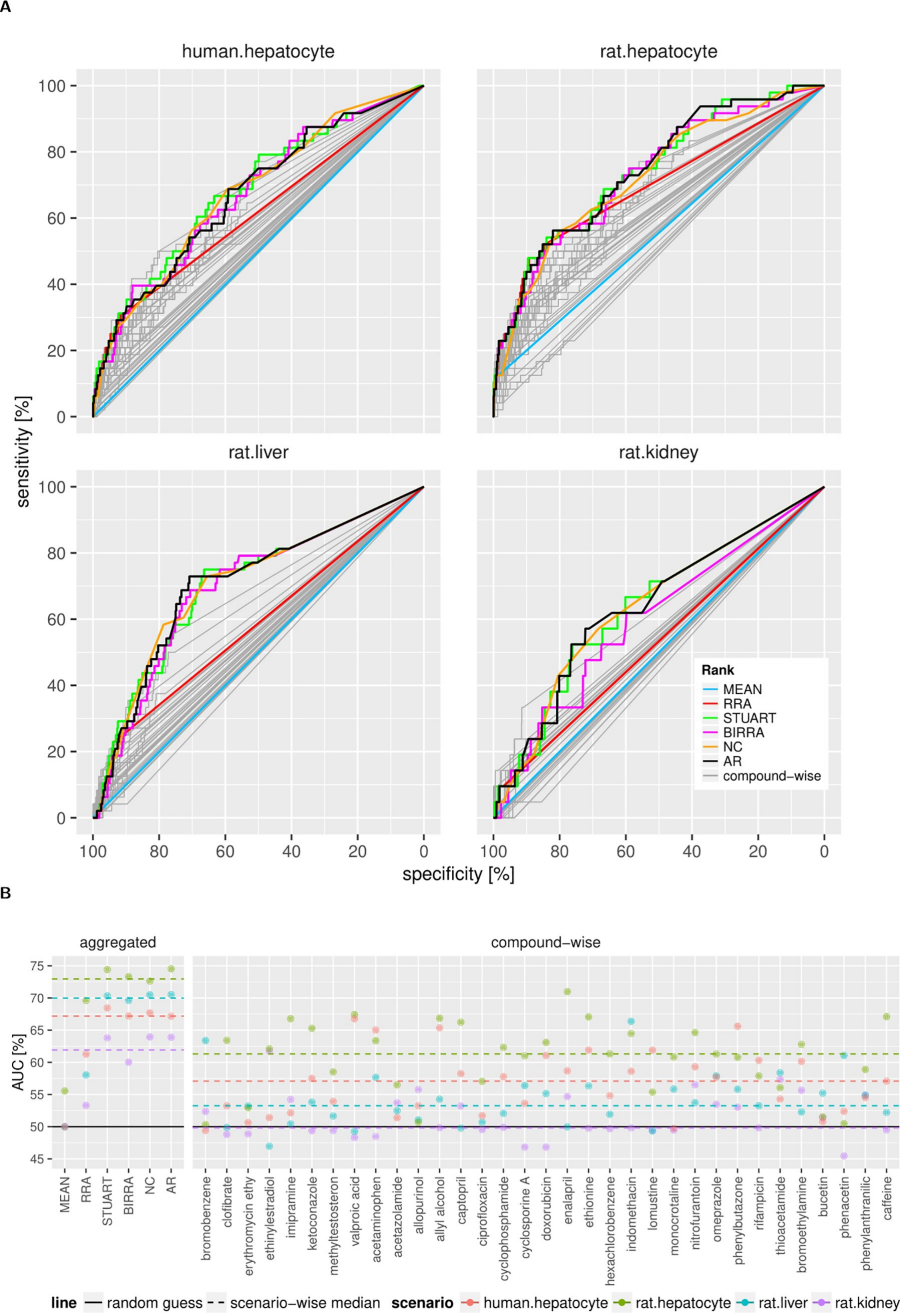
Performance comparison of gene ranking methods. A) ROC curves for compound-wise rankings (gray curves) and compound-aggregated rankings (coloured curves) as calculated by the rank-aggregation methods, with literature-derived genes used as positive examples. Curves are not smoothed, and the straight line fragments of the curves result from the initial partial ranking (same lowest rank for all not-modulated genes, see Methods for details). B) Summary of AUC (area under the ROC curve) values for all rankings across all model systems, including model system-wise median AUC represented as dashed line. AUC values above 50% means better performance than randomly assigned rank.

The ROC evaluation of the aggregation methods (Fig 2A, S3 Fig) across compounds suggests that the rankings from models with largest numbers of differentially expressed genes recapitulate better the genes from the literature. Overall, in the *in vitro* scenario we get better performance (larger AUC) as compared to the *in vivo* one. While the highly imbalanced size of differentially expressed gene sets seems to affect the MEAN aggregation method the most, the NC rank, which simply uses the number of compounds modulating a given gene, works in par with the more complex aggregation methods. Due to the influence of the partial ranking, the MEAN method and to a lesser extent the RRA method delivered often the same ranks for the larger sets of genes; at the same time, the NC rank was quite high for these. The latter indicated that we might benefit if we do second-level aggregation across the rank aggregation methods, and one simple way is to go for the median of the aggregated ranks (named AR).

Evaluation of aggregation methods indicated that STUART was the optimal with AR very close (Fig 2B). Since AR is an aggregation method, we preferred it for the final gene prioritization. We selected the top 400 AR-ranked genes in each scenario for down-stream analysis.

The four rankings reflect how the four systems (HH, RH, RL, RK) respond to the same set of toxic compounds and thus allow us to evaluate their strengths and their limitations in relation to each other in terms of recapitulating different aspects of human organ toxicity as reflected in affected gene expression. The connection to genes allows the generation of hypotheses about the concrete biological processes involved.

**Fig 3:**
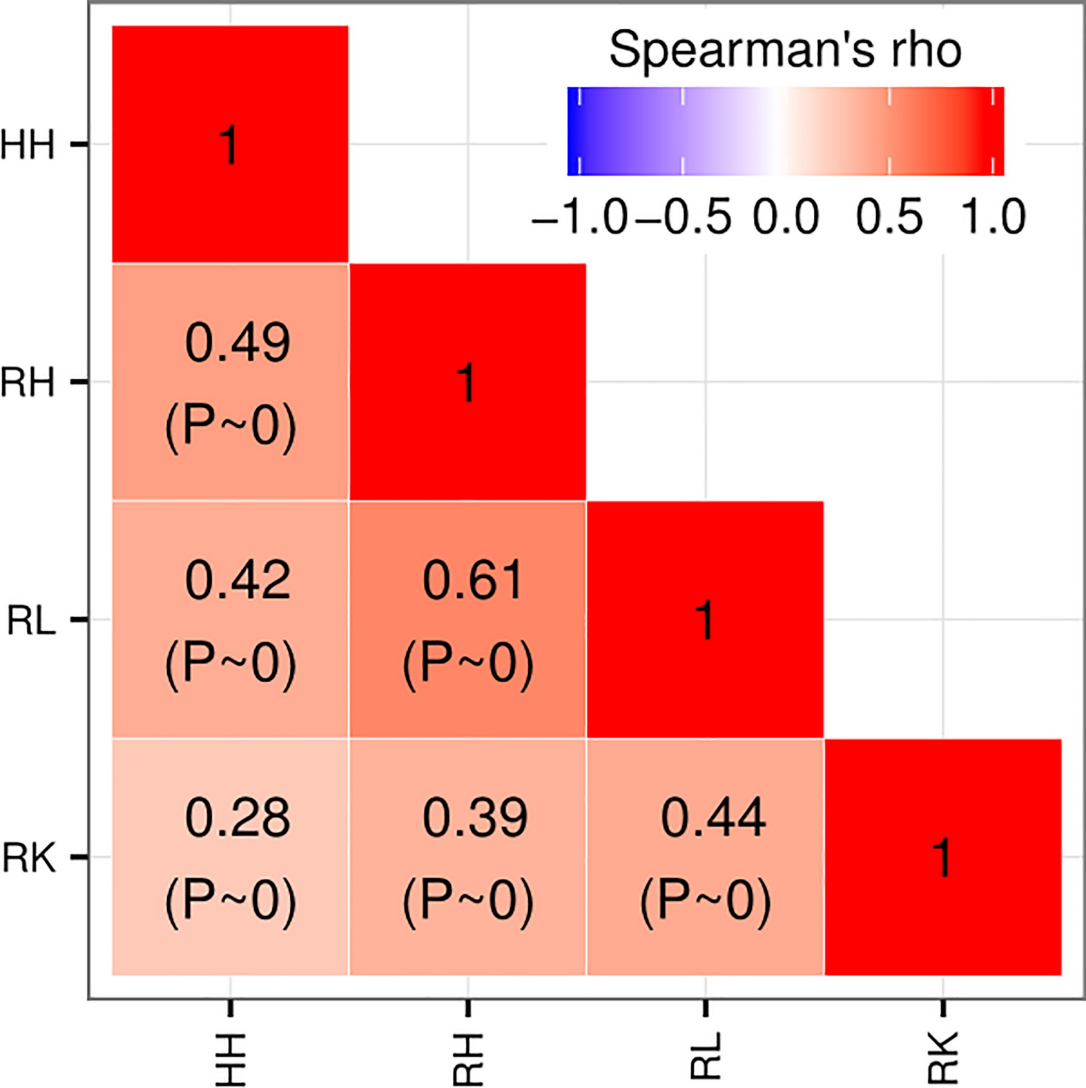
Comparison of AR-ranked gene sets across model systems. Rank correlation between the model system-specific gene rankings as measured by the Spearman’s rho correlation coefficient.

Examination of the highest 10 ranked genes (Table 1, columns HH, RH, RL, RK) shows no overlap between models. A more meaningful result is obtained when fully comparing the four rankings (Fig 3): the best correlation found is between RH and RL (Spearman’s rho = 0.61). RH to HH is second best (Spearman’s rho = 0.49), which suggest that they certainly differ in terms of response to toxic drugs, but not so much than when comparing other models (e.g., RK versus RL, Spearman’s rho = 0.28).

**Table 1:** Top 10 ranking genes for each model (HH, RH, RL and RK) and for combined rankings: Sum (all), and subtractions (HH>RH, RH>HH, RL>RH and RH>RL). Font indicates toxicity association by literature, bold for liver, underlined for kidney.

HH	RH	RL	RK	all	HH>RH	RH>HH	RL>RH	RH>RL
GDF15	SELENBP1	CGN	TRIM35	**HMOX1**	BCHE	**GSTP1**	RPL14	SOX4
PDZK1IP1	**CYP2C19**	MAT1A	DNAJB9	ZFAND2A	SERPINA5	IDH1	DSP	ID3
UPP1	C4BPB	KIAA0101	**MTHFR**	KIAA0101	MCHR1	ACTA1	CCNL2	AGXT2
NUSAP1	**CYP1A2**	INHBA	USP2	SRXN1	TMEM138	CYP26B1	ACKR4	IDH1
PCK1	NNT	TUBB	IFRD2	HERPUD1	EFEMP1	SLC6A13	SLC25A32	CD276
DDB2	OTC	UBE2C	HSPA12A	CDK1	HNMT	PSCA	SLC16A1	ACTA1
SLC2A2	FBP1	TMCC3	EIF2S2	CCNA2	LARP6	RBP4	CTPS1	FAM111A
FSTL1	**GSTP1**	SPC25	SLC17A3	UBE2C	HADHA	NXPE4	NIM1K	CD14
TYMS	PKLR	TOP2A	MAFF	KIF20A	C16orf45	AQP8	ABCB9	GC
**HMOX1**	AMPD3	CDO1	TMEM37	HMGCS2	PALM3	PXDC1	LIMS1	MAN2A1

To find out about the processes affected by toxicity in each of the four models, we did functional enrichment analysis of various terms for which the genes were annotated (including Gene Ontology terms, pathways, participation in protein complexes, and tissue expression; see Methods for details).

Interestingly, now the similarity between the top-100 genes of HH and RH is manifested in terms of functional GO BP (Gene Ontology Biological Process) terms, where the most significant are “carboxylic acid metabolic process” and “monocarboxylic acid metabolic process”, respectively (see S5 and S6 Files). In both systems “response to drug” appears, although at a less significant level.

A similar analysis for the top-100 genes of RL indicates terms related to cell cycle and cell division (S7 File) indicating that the processes most affected are less specific when studying an organ. In the case of RK we did not even obtain terms at high significance level (P-value < 1e-03; S8 File).

### Combining the ranked gene lists for the four model systems

While the functional analysis above informs of the processes affected by toxicity in each of the four models, we assume that the rankings can be meaningfully combined to better learn about the similarities and differences between models. In the following paragraphs, we illustrate how we can do this comparison (S4 File) and discuss the insight that can be gained.

#### Genes dys-regulated in the four model systems

To study genes dys-regulated in the four systems we added the four rankings (column “all” in S4 File) and sorted the genes by value (see top 10 genes in Table 1, column “all”). We would claim that these genes have functions consistently related to toxicity because they are high in this aggregated ranking.

The top gene is HMOX1, selected from both of our literature analyses as kidney and liver toxicity related. Other top genes include zinc finger, AN1-type domain 4 (ZFAND2A), PCNA clamp associated factor (KIAA0101, now known as PCLAF or p15PAF), sulfiredoxin 1 (SRXN1; the only gene that appeared among the top 400 genes of each individual AR-ranked list) and homocysteine-inducible, endoplasmic reticulum stress-inducible, ubiquitin-like domain member 1 (HERPUD1). Both SRXN1 and HMOX1 are direct targets of the nuclear factor, erythroid 2-like 2 (NFE2L2, also known as NRF2; which ranked among the top 400 genes in the three rat model systems, but very low in the human hepatocytes), a transcription factor important for the coordinated up-regulation of genes in response to oxidative stress [40]. Overexpression of KIAA0101 is associated to poor prognosis in several cancers, e.g., hepatic, primary lung [41], gastric [42], and esophageal [43]. The protein encoded by this gene binds both to PCNA and to DNA and thus is assumed to have a function in the control of DNA replication and repair [44].

The next two genes are cyclin-dependent kinase CDK1 and cyclin CCNA2 (see Table 1), and in fact many cyclins and cyclin-dependent kinases are observed among the top 100 genes (see S4 File). Using functional enrichment as above, confirmed cell cycle related enrichment (S9 File). The most significant term for the GO BP category was “mitotic cell cycle” (22 genes of top 62; P-value = 1.87e-12), “Cell cycle kinase complex CDC2” for CORUM (all 6 genes of this category found among the top 89 in the list), “Cell cycle” for KEGG, and “Cell Cycle” for REACTOME. The BP GO term “response to toxic substance” was highly significant too (16 genes among the top 65; P-value = 1.72e-08), followed close by the more general term “cellular response to chemical stimulus” to which half the genes were related (40 genes of top 88; P-value = 1.41e-07).

Terms explicitly related to response to drugs were found enriched only in HH and RH and with worse P-values (“response to toxic substance”, 1.18e-05 and 7.8e-06, respectively). Collectively, we took these results as an indication that this way of aggregating the ranks was useful to point to genes and processes generally associated to toxicity in liver and kidney.

#### Human versus rat hepatocytes

To evaluate differences between the two *in vitro* systems, i.e., HH versus RH, we re-ranked the genes by a simple score calculated by subtracting the RH rank from the HH rank (column “HH vs RH” in S4 File). A low value for the resulting score highlights genes that changed in HH and not in RH, while a high value means the converse. The top genes influenced by toxicity in HH but not in RH were BCHE, SERPINA5, MCHR1 and TMEM38 (see column “HH>RH” in Table 1). While this could be due to differences in the functions of these proteins, which could be reflected by differences in their sequences, this was not always the case: TMEM138, transmembrane protein 138, is highly conserved between rat and human (91% sequence identity), and so is BCHE, butyrylcholinesterase (79% sequence identity).

At the other side of the list, we can see the genes that were highly affected by drugs in rat hepatocytes but not in human: glutathione S-transferase pi 1 (GSTP1) and isocitrate dehydrogenase (NADP+) 1 (IDH1) are at the top (column “RH>HH” in Table 1). The human IDH1is 100% identical to the rat ortholog. The case of GSTP1 is intriguing since both proteins in rat and human are 210 amino acids long of which 180 are identical (85% sequence identity): the human ortholog was not found to be differentially expressed in any of the 33 compounds for the high dose case, while in rat hepatocytes it was found in 23 cases. Similarly, IDH1 was selected as differentially expressed by 0 and 22 compounds for human and rat hepatocytes, respectively. All these pairs of orthologs seem to have very similar lengths, and possibly domain architectures. We conclude that the list indicates physiological differences.

Functional enrichment analysis did not suggest functions associated to these differences for the top 100 genes affected in HH but not in RH (S10 File). Differently, the top 100 genes affected in RH and not in HH were enriched in genes annotated with GO CC “extracellular exosome” (30 of top 97; P-value = 1.67e-04), which is a very general category, and intriguingly, the most significant GO BP was “retinoid metabolic process” (with 6 genes among top 84; P-value = 3.86e-03), among them CYP26B1 and RBP4, which are among the top 10 (Table 1; S11 File).

#### Rat liver versus rat hepatocyte

Finally, we checked what different toxic responses we see when looking for genes affected by drugs in rat liver but not in rat hepatocytes. For this purpose, we compared RL with RH by subtracting the RH rank from the RL rank (see column “RL vs RH” in S4 File) and re-ranking the genes by the resulting score (column “RL>RH” in Table 1). Functional analysis indicated mostly genes expressed in glandular tissues (hpa terms; top one is “endometrium; glandular cells”, 30 genes among top 73, P-value = 1.76e-04), and the most significantly enriched GO BP term was “localization”, which refers to transport process (S12 File). These genes would reflect processes involved in liver toxicity outside the hepatocyte.

At the other side of the list, we observed genes that could reflect hepatocyte-specific toxicity mechanisms. As genes changing most in RH and less in RL we observed SOX4 and ID3 (Table 1). SOX4, sex determining region Y-box 4, is a transcription factor involved in the regulation of embryonic development and in the determination of the cell fate. While this factor is absent in normal adult liver, it has been recently shown that it plays an important function in liver tumor metastasis [45]. ID3, inhibitor of DNA binding 3, dominant negative helix-loop-helix protein, and other genes in the ID family have been noted to be down-regulated by iron particles in hepatocytes [46]. Functional enrichment analysis indicated enrichment in genes with GO CC term”extracellular exosome” (27 genes of top 63; P-value = 1.90e-07), but may be more meaningfully, the most significant GO MF term was “lipid binding” (8 genes of top 19) and two of the top 4 genes have the GO BP term “glioxylate metabolism” (AGXT2 and IDH1), functions characteristic of the hepatocyte (S13 File).

In summary, S5 File collects the full ranks for the four models and allows the comparison of toxicity responses in the four model systems considered. Scoring and sorting the genes combined with enrichment analysis can be a powerful strategy to pinpoint differences and similarities of toxicity responses in the four scenarios.

## Discussion

Here, we presented a protocol to extract ranked gene lists from toxicogenomics datasets specific to model systems (e.g. human hepatocytes), which includes evaluation by agreement with the literature in human toxicity (e.g. dealing with human hepatotoxic genes). This approach allows evaluating different ranking methods by the agreement of the resulting gene rankings to the literature, and can be used to evaluate and compare the aspects of human toxicity recapitulated by each system model.

Due to the multi-factorial experimental designs of the existing toxicological databases, we needed to integrate data across multiple model systems, drugs, dose levels and time points. A relatively simple way is to summarize the genome-wide drug-induced transcriptional changes in a prioritized (ranked) gene list, followed by robust rank-aggregation approach [15] to combine them into a single organ-specific gene list. While this approach is commonly used for prioritization of disease-related genes [14], here we used it as baseline method for prioritization of toxicity-related genes. In particular, we exploit the robustness (with respect to noise, outliers, errors) and computational efficiency of recently proposed probabilistic models for rank-aggregation, i.e., RRA [15] and BIRRA [16].

While the task of aggregating results from toxicogenomics datasets is clearly not trivial [3], current research efforts show that carefully designed protocols for meta-analysis can potentially address issues of heterogeneity of samples. For example, novel (more generic) gene biomarkers for drug-induced toxicity have been identified by integration of differential expression analysis and data mining of *in vitro* and/or *in vivo* toxicogenomics data [47, 48]. Several studies exploited biclustering methods [49] for coherent coexpression gene module discovery combined with downstream functional enrichment analysis to characterize conserved pattern of drug response across human cell lines [50] and prioritize gene modules for specific chemically-induced liver [51] and kidney injury endpoints [52]. Last, but not least, El-Hachem et al. [53] showed that mapping the drug-induced transcriptional changes in hepatocytes into the space of enriched biochemical pathways, combined with subsequent bi-clustering, reveals conserved modules of chemically modulated pathways in human hepatocytes, rat hepatocytes and rat liver model systems.

Here, we focused on the evaluation of organ-specific toxicity-associated genes, and proposed a protocol to prioritize genes that we evaluated with the literature data, with a focus on kidney and liver. We applied this approach to a toxicogenomics dataset corresponding to 33 compounds (mainly toxic drugs) profiled in rat kidney (RK), rat liver (RL), primary rat and human hepatocytes (RH and HH, respectively), obtained from Open TG-GATEs. This resource was especially fitting our purpose, due to consistent experimental design across the four target systems; TG-GATEs has been recently exploited to find hepatotoxic compounds [54] and to compare rat liver testing systems [55]. We evaluated multiple ways to aggregate these data for the description of drug-induced toxicity in human kidney and liver.

In our analysis, we chose maSigPro for modeling gene expression as it perfectly fits the dynamic nature of the data (multiple time series). On the other hand, maSigPro selects significantly different gene expression profiles based on two-step approach, in which genes that do not pass the first step (for a given significance) will not be modeled in the next step (no P-value and R2 calculated), meaning a worst possible rank in our case. This means that the compound-wise gene lists are technically partially ranked lists. While this can be a problem for standard rank-aggregation methods (as evident for the mean rank in the ROC analysis), the alternative approaches RRA, Stuart and BIRRA were not specifically affected, making the proposed method suitable for meta-analysis of partially ranked gene lists across compounds obtained from literature.

There is a substantial level of gene filtering in our protocol, which was necessary to fairly compare the rank-aggregation methods across species and with the literature-derived human gene lists. The current method uses only one-to-one orthologs as defined in NCBI Homologene databases. Furthermore, the current approach for generating toxicity-related genes from annotated biomedical literature takes only into account human protein-coding genes. This means that the results should improve by inclusion of more orthology relations (available in other databases) as well as by extension of the literature-derived lists with toxicity related non-coding human genes.

The differential expression analysis as well as the rank-aggregation analysis across drugs show that in the *in vitro* model systems we get better coverage of the human gene candidates from the literature as compared to the *in vivo* one. The highly imbalanced size of the sets of differentially expressed genes seems to affect the mean aggregation method the most. Despite this, aggregation of partial rankings is still capable to find common drug-response genes across all model systems. An additional factor that could explain the better agreement to the *in vitro* models is the fact that toxicity is often tested in cell lines and not *in vivo*. For example, around a 30% of references related to the toxicity genes ATF3, GDF15, HMOX1 and EGR1 mentions also a cell line.

Another factor that could influence the differences between the ranks derived from the four model systems for their agreement with the literature-derived lists could lie in the differences in numbers of genes associated in the literature (which itself could reflect real physiological differences). Accordingly, the lowest agreement found in RK could be related to the fact that the number of literature-derived genes associated with kidney toxicity was much smaller than those associated to liver toxicity. Both the HH and RH system recapitulated better the literature than RL, maybe indicating that the *in vitro* systems are more appropriate in general to model liver toxicity. Unexpectedly, RH was a bit better than HH: while the similar performance indicates that RH is a good model for studying liver toxicity, this result could be influenced by the fact that studies dealing with human genes (among the selected PubMed abstracts used for extraction of the literature-derived gene lists) might actually use rodent models.

Ranked lists for the four models, *in vitro* HH and RH, and *in vivo* RL and RK, indicated little agreement in the top ranked genes (Table 1). Full rank comparisons (Fig 3) suggested that the two rat hepatic models, RH and RL, are the most similar, while the two *in vitro* hepatocyte models (HH and RH) follow next. Functional analysis, however, suggested that HH and RH were the models more similar in terms of the functions of genes affected, mostly related to carboxylic acid metabolism.

Next, we combined the ranks looking for genes ranking high in the four models. Adding pragmatically all ranks brings HMOX1, a very well-known toxicity associated gene to the top position, and highlights a significant number of genes related to responses to toxicity and chemical stimuli, suggesting that the addition of rankings is meaningful. Collectively, cell cycle and cell division seem to be the processes most generally affected in the models. Rank comparison between HH and RH highlights genes with astonishing different reactions to toxicity. This suggest that researchers should not use RH as toxicity model if they are interested in certain genes related to human liver toxicity that react in HH but are non-responders in RH, such as GSTP1. Similarly, our comparison of RH and RL indicates that while researchers interested on toxic effects of genes related to lipid binding should be using hepatocytes, important toxic effects on transporters that are observed in RL will be missed if using RH.

As every integrative approach that relies on multiple subsequent steps of data preprocessing, modeling, and high-level summarization, our integration protocol can be influenced by the limitations of each subsequent step of the analysis: from comprehensiveness of the input (toxicogenomics) data, issues with data normalization and gene expression modeling, to specificity and sensitiveness of the method (maSigPro) to detected gene expression changes, the criteria used for ranking (P-value and R2 of the gene-wise models) as well as the robustness of the subsequent aggregation of gene lists (median of ranks, rank sum). However, the proposed protocol is fully flexible in the sense that each of the subsequent steps can be easily replaced with suitable alternatives.

Our approach to find literature derived genes also has some limitations. For example, we missed PON1, which is known to be related to liver toxicity (see e.g., [56]). The reason is that we used a conservative approach with stringent cut-offs in the selection of positives (see Methods for details).

Here we have presented a protocol for the aggregation and literature evaluation of toxicogenomics data. Application to liver and kidney model systems of *in vitro* and *in vivo* toxicity allowed us to define genes and pathways with general and particular toxicity related roles in these models. We believe that this protocol should be easily applicable to other human organs and to other models in toxicogenomics datasets.

## Supporting information

S1 Fig Dose-dependent summary of differential expression analysis. The distributions of the number of differentially expressed genes are depicted with violin-plots by model systems, with each dot representing the number of genes modulated by a single compound. Each row corresponds to one dose level. The median and interquartile range of each distribution is depicted by black dot and black bars respectively. Left column: full set of genes. Middle column: genes with rat ortholog. Right column: literature-derived genes.
(TIFF)

S2 Fig Compound-specific distribution of differentially expressed gene set sizes. Each plot corresponds to one compound (only high dose treatments results), with each dot corresponding to the number of differentially expressed genes (all) obtained in a single model system including the subset of genes having a rat otholog (othologs) and the subset found in the literature-derived lists (literature). The plots are grouped by the toxicity label of the compound: hepatotoxic in the first row, toxic for both organs in the second and the third row, and in the last row we have few nephrotoxic and caffeine, not classified as a toxic compound.
(TIFF)

S3 Fig Compound-specific AUC (ROC) performance across model systems. Each plot represents a single compound, depicting ROC curves for (compound-specific) model system-specific gene rankings (each color represents one model system), with literature-derived genes used as positive examples. Curves are not smoothed, and the straight line fragments of the curves result from the partial ranking (same lowest rank for all not-modulated genes, see Methods for details). AUC (area under the ROC curve) values above 50% means better performance than randomly assigned rank. The plots are grouped by the toxicity label of the compound: hepatotoxic in the first row, toxic for both organs in the second and third row, and in the last row we have few nephrotoxic and caffeine, not classified as a toxic compound.
(TIFF)

S1 File Compounds and data samples summary. Excel file with description of the 33 compounds profiled across all model systems in Open TG-GATEs database. The file includes full and abbreviated names, and if available the toxicity labels, compound IDs in on-line compound databases (PubChem and DrugBank), chemical structure description (by SMILES), mechanism, mode of action as well as therapeutic labels. The file also includes a description of the array data used.
(XLSX)

S2 File Literature-derived genes. Excel file with full lists of literature-derived organ-specific toxicity-related genes, including the output of the co-occurrence analysis as well as the disease enrichment of the organ-specific lists.
(XLSX)

S3 File Gene rankings. Excel file with all gene rankings (obtained by the different ranking methods) for each model system in a separate data sheet. All data sheets are ranked according to the finally chosen AR-ranking. The gene Entrez IDs and gene symbols correspond to the human annotation space. Note that this file uses compound abbreviations introduced in S2 File.
(XLSX)

S4 File Aggregation of AR ranks. Excel file with full gene list ranked according to the sum of all model system-specific AR ranks. The gene Entrez IDs and gene symbols correspond to the human annotation space. Extra columns include combinations of the AR rankings needed for comparison between the modeled systems.
(XLS)

S5 File Functional enrichment analysis of the top-100 HH ranked genes. Text file with the output of a functional term enrichment analysis using the g:Profiler web tool for the top-100 HH ranked genes.
(TXT)

S6 File Functional enrichment analysis of the top-100 RH ranked genes. Text file with the output of a functional term enrichment analysis using the g:Profiler web tool for the top-100 RH ranked genes.
(TXT)

S7 File Functional enrichment analysis of the top-100 RL ranked genes. Text file with the output of a functional term enrichment analysis using the g:Profiler web tool for the top-100 RL ranked genes.
(TXT)

S8 File Functional enrichment analysis of the top-100 RK ranked genes. Text file with the output of a functional term enrichment analysis using the g:Profiler web tool for the top-100 RK ranked genes.
(TXT)

S9 File Functional enrichment analysis for genes dys-regulated in the four model systems. Text file with the output of a functional term enrichment analysis using the g:Profiler web tool for the top-100 ranked genes according to the sum of the four rankings: HH, RH, RL, RK.
(TXT)

S10 File Functional enrichment analysis for genes dys-regulated in human hepatocytes but not in rat hepatocytes. Text file with the output of a functional term enrichment analysis using the g:Profiler web tool for the top-100 ranked genes according to the difference between the HH and the RH ranking.
(TXT)

S11 File Functional enrichment analysis for genes dys-regulated in rat hepatocytes but not in human hepatocytes. Text file with the output of a functional term enrichment analysis using the g:Profiler web tool for the top-100 ranked genes according to the difference between the RH and the HH ranking.
(TXT)

S12 File Functional enrichment analysis for genes dys-regulated in rat liver but not in rat hepatocytes. Text file with the output of a functional term enrichment analysis using the g:Profiler web tool for the top-100 ranked genes according to the difference between the RL and the RH ranking.
(TXT)

S13 File Functional enrichment analysis for genes dys-regulated in rat hepatocytes but not in rat liver. Text file with the output of a functional term enrichment analysis using the g:Profiler web tool for the top-100 ranked genes according to the difference between the RH and the RL ranking.
(TXT)
