# Supplementary figures and images for: Literature optimized integration of gene expression for organ-specific evaluation of toxicogenomics datasets
Source: PLoS One. 2019 Jan 14;14(1):e0210467. doi: 10.1371/journal.pone.0210467 (PMC6331104; doi:10.1371/journal.pone.0210467)

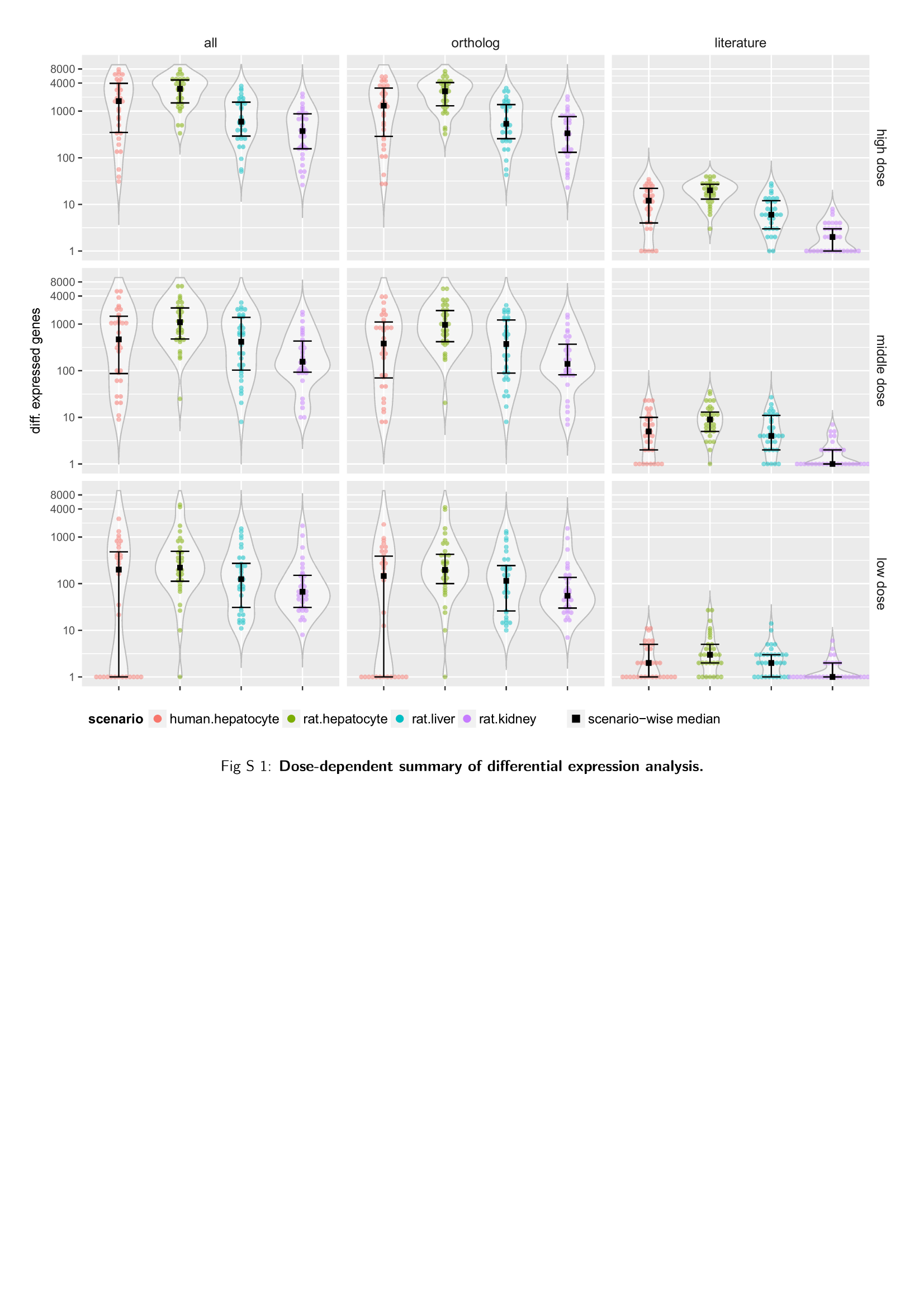

Supplement: S1 Fig — The distributions of the number of differentially expressed genes are depicted with violin-plots by model systems, with each dot representing the number of genes modulated by a single compound. Each row corresponds to one dose level. The median and interquartile range of each distribution is depicted by black dot and black bars respectively. Left column: full set of genes. Middle column: genes with rat ortholog. Right column: literature-derived genes. (TIFF) [file pone.0210467.s001.tiff]

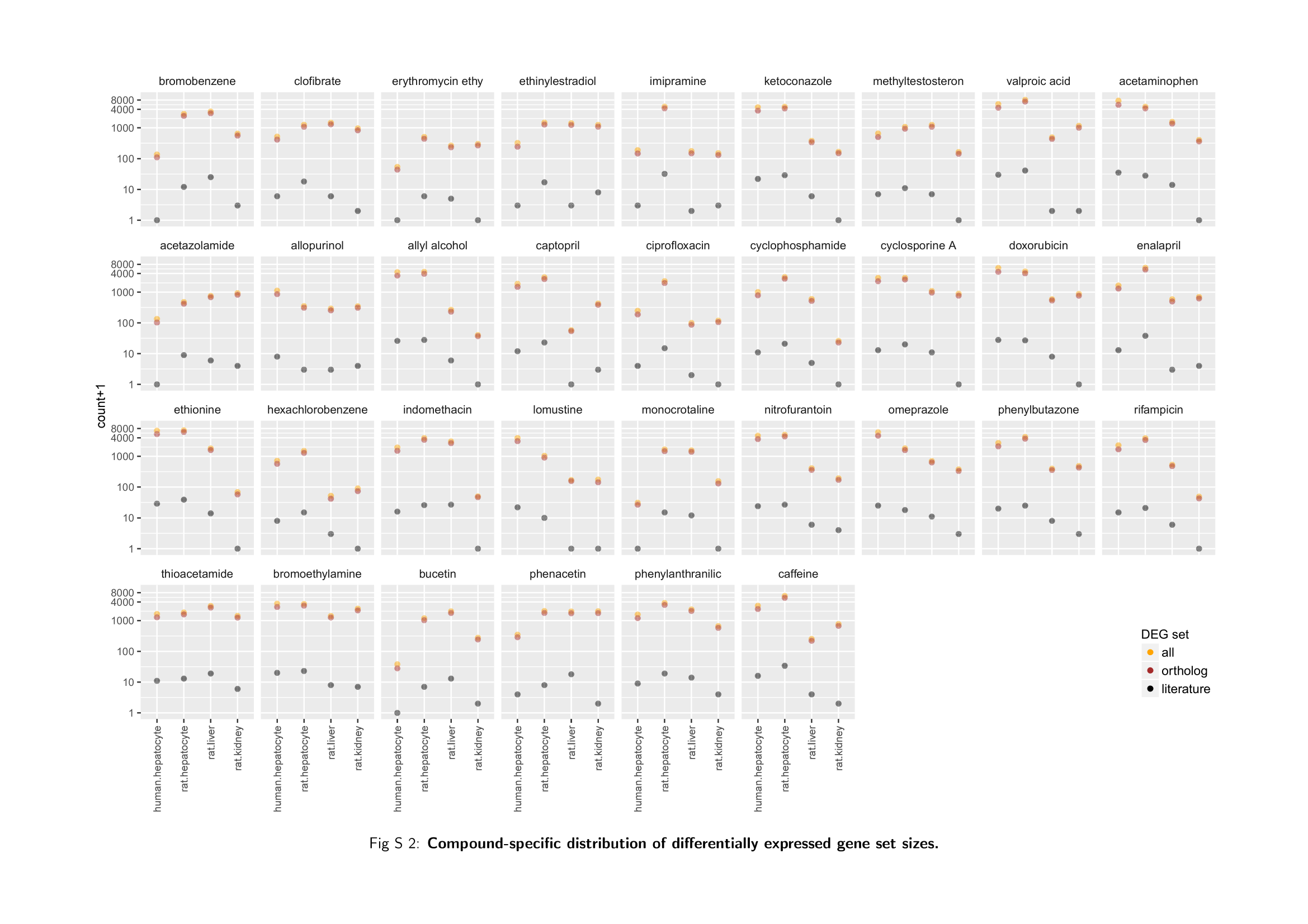

Supplement: S2 Fig — Each plot corresponds to one compound (only high dose treatments results), with each dot corresponding to the number of differentially expressed genes (all) obtained in a single model system including the subset of genes having a rat otholog (othologs) and the subset found in the literature-derived lists (literature). The plots are grouped by the toxicity label of the compound: hepatotoxic in the first row, toxic for both organs in the second and the third row, and in the last row we have few nephrotoxic and caffeine, not classified as a toxic compound. (TIFF) [file pone.0210467.s002.tiff]

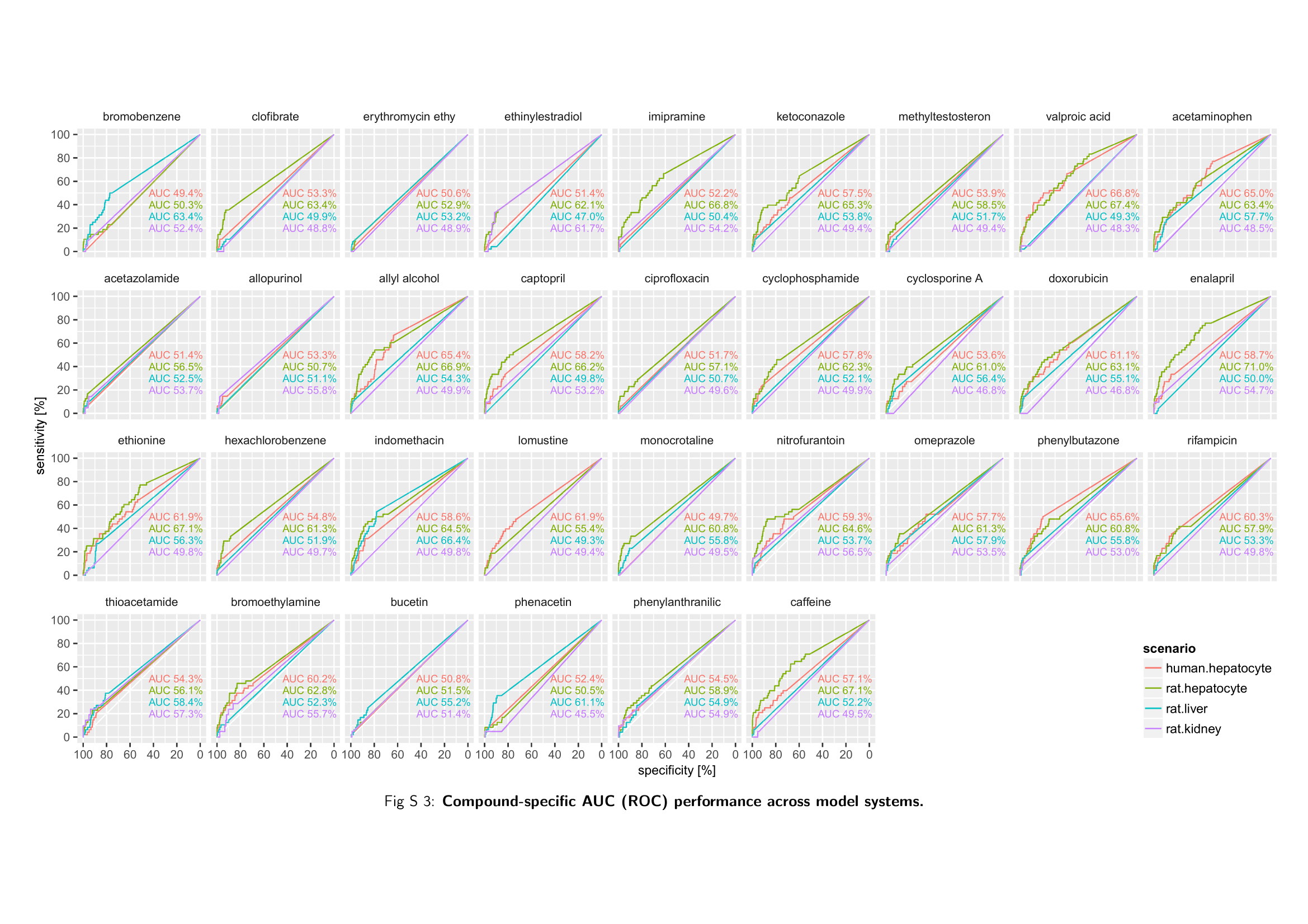

Supplement: S3 Fig — Each plot represents a single compound, depicting ROC curves for (compound-specific) model system-specific gene rankings (each color represents one model system), with literature-derived genes used as positive examples. Curves are not smoothed, and the straight line fragments of the curves result from the partial ranking (same lowest rank for all not-modulated genes, see Methods for details). AUC (area under the ROC curve) values above 50% means better performance than randomly assigned rank. The plots are grouped by the toxicity label of the compound: hepatotoxic in the first row, toxic for both organs in the second and third row, and in the last row we have few nephrotoxic and caffeine, not classified as a toxic compound. (TIFF) [file pone.0210467.s003.tiff]
